# Introduction from Guest Editors to Special Issue “Magnetism in Chemistry”

**DOI:** 10.3390/ijms24020899

**Published:** 2023-01-04

**Authors:** Mauro Fianchini, Jose Gracia, Oleg V. Mikhailov

**Affiliations:** 1 MagnetoCat SL Calle General Polavieja 9 3l, 03012 Alicante, Spain; 2 Department of Analytical Chemistry, Certification and Quality Management, Kazan National Research Technological University, K. Marx Street 68, 420015 Kazan, Russia

Magnetism in a chemical compound is the macroscopic result of the couplings of the spins of electrons within in it. The spin is the intrinsic magnetic moment of an electron, our awareness of which rises from the combination of the most important theories of the 20th century: quantum mechanics and relativity. The spin has no counterpart in classical physics, although a nice analogy that could at least approximate its conceptual visualization is the angular moment vector generated in the rotation of a spin-top: the spin-top may “spin” clockwise or anticlockwise and the angular momentum vector, m$\vec{v}$x$\vec{r}$, will consequently point inward or outward, respectively. Particles such as electrons obey to Fermi–Dirac statistics are, therefore, called fermions. The fermions have half-integer spin values, such as 1/2, 3/2, 5/2, etc. (in the case that the electron is 1/2 in a quantized ℏ unit, Planck’s constant). 

The history of the “quantized spinning” of the electron starts with A. K. Compton, who suggested this concept in 1921 as a way to conceive of the idea of a natural unit of magnetism. In 1922, O. Stern and W. Gerlach demonstrated the existence of a quantized magnetic moment in atoms in a very famous experiment, where a narrow beam of silver atoms was fired towards a target through a vertical magnetic field; two spots were noted on the target, demonstrating the incontrovertible existence of two possible orientations for the intrinsic magnetic moment of the electron. T.E. Phipps and J.B. Taylor confirmed the Stern–Gerlach experiment in 1927, using hydrogen in its ground state to avoid any possible claim that the silver may have tampered with the correct interpretation of the experiment. S. Goudsmit and G. Uhlenbeck applied the concept of the “spinning electron” to interpret aspects of the quantum treatment of the Zeeman effect and complex spectra in general, and they proposed the existence of a fourth quantum number, the spin quantum number. W. Pauli introduced the quantomechanic treatment of spin using the matrices that bears his name: the matrices were introduced into the non-relativistic Schrodinger equation to correct its false prediction of a null magnetic moment for hydrogen atoms. Pauli matrices were later re-used in the relativistic treatment of the Schrodinger equation proposed P. Dirac. Dirac’s spinors implemented two extra degrees of freedom related to the spin into the relativistic Klein–Gordon equation.

In complex multi-atomic structures, the spins cooperate amongst each other to form ferromagnetic, ferrimagnetic, or diverse antiferromagnetic orderings below the Curie Temperature, or in general paramagnetic states above this temperature. In ferromagnetic domains, the spins of the unpaired electrons are parallel to each other, but not necessarily in a precise direction of space. Electrons in antiferromagnetic couplings exist in an anti-parallel, inter-atomic fashion, with a zero net magnetization result. Many types of antiferromagnetism exist, from the most common, such as the A-, C-, and G-types, to more complex examples, such as the E- and E’-types. An important aspect is that in all cases except AFM type G, the spins create ferromagnetic channels in one or two directions of space. 

The quantum exchange mechanism is responsible for the magnetic orderings associated with open-shells. Quantum exchange is a scattering mechanism responsible for the exchange of the confining potential (orbital) between two electrons possessing parallel spins in two different ϕ and χ orbitals, capable of reducing their mutual Coulomb repulsion. However, it is not the only one: another scattering mechanism allows for two electrons with anti-parallel spins to “jump” to empty orbitals; this mechanism also reduces Coulomb repulsions between the electrons because the vertical excitation of the electrons place them in more diffused potentials in space and temporarily farther away from each other. The stabilizing quantum spin exchange mechanism leads to magnetic configurations that are crucial to generate dominant ferromagnetic spin channels and conductivity (the promotion of electrons to excited orbitals in solids means the population of the conduction band) in catalysts. Recent have studies demonstrated, in fact, that itinerant ferromagnetic spin channels (in at least one direction of space) are prerequisites to achieving excellent catalytic performances in crucial non-conserving spin reactions. Such reactions can be used, in particular, for the production of so-called “green hydrogen”, the destruction of water (in which a closed-shell species such as H_2_O is transformed into an open-shell ^3^O_2_) and hydrogen oxidation (where the open-shell species ^3^O_2_ is transformed into diamagnetic H_2_O).

Magnetic couplings, or spin potentials, are the quantum interactions in homogeneous or heterogeneous catalysts that can be properly tuned to improve catalysed chemical reactions by boosting kinetic aspects (e.g., determining the acceleration of the reaction rate), thermodynamic aspects (e.g., stabilizing/destabilizing physisorption and chemisorption of reactants, products, and/or intermediates onto the catalytic species or surfaces), or transport phenomena (e.g., diffusion, solvation). The relevance of this topic is growing so much nowadays that a new branch of electro-catalysis may even be introduced by the name of “spintro-catalysis.” Spin-catalysis in general is a type of catalysis, influenced, tuned, and improved by adjusting the electronic spins as well as the intrinsic magnetic domains of the active sites. The catalyst can also be magnetized using external magnetic fields (extrinsic magnetism). In such case, the presence of an external direct spin potential favours the polarization of the electrons and the alignment of the possible magnetic domains. The external field may then be removed (if the catalyst does not demagnetize) or maintained (to avoid the decay of the magnetization) during the catalytic event. Many authors reported improvement in the kinetics of several catalyses by 10–20% via the use of magnetic catalysts and/or external magnetic fields.

Magnetic catalysts typically contain open-shell metals from the *d*-block and/or the *f*-block, thus being part of the strongly correlated structural fragments of chemical compounds. The first series includes metals such as V, Mn, Cr, Fe, Co, and Ni. The catalytic behaviours of compounds containing magnetic metals usually fail to be captured via widespread approximations employed for heterogeneous catalysis. A.A. Balandin, who studied the chemical and physical effects that affect heterogeneous catalysis by introducing “volcano” plots as a useful tool, noticed that a strong correlation exists between the bonding energy of certain metals with hydrogen and their magnetic states. Recent studies demonstrated that trends versus the atomic number involving magnetic catalysts cannot be described by simple “volcano” plots but rather by more complex multipeak plots. 

Much scientific interest has also been raised around heterogeneous (including single-atom) and homogeneous magnetic catalysis in the last decade because of the capability of magnetic catalysts to break the symmetry in spin-forbidden reactions and accelerate them more proficiently than their closed-shell counterparts. Two notably famous reactions that do not conserve spin are the oxygen evolution reaction and the oxygen reduction reaction. OER and ORR are crucial reactions for the production and the use of “green” hydrogen as source of clean, renewable, and sustainable energy. The production of hydrogen can be carried out via water splitting, a chemical reaction where water is divided into its basic components, hydrogen (H_2_) and oxygen (^3^O_2_). Water splitting can be divided further into two half-reactions (usually happening at different electrochemical half-cells): the hydrogen evolution reaction (HER) (happening at the cathode), and the oxygen evolution reaction (OER) (happening at the anode). An electrical current is needed to carry this process to completion. The energy should ideally be produced by renewable energy sources, such as photovoltaic energy, for instance; this methodology should lower the environmental footprint and decrease, if not nearly curbing, the emissions of greenhouse gases, sulphur oxides, nitrogen oxides, and other contaminants deriving from the combustion of fossil fuels. Oxidizing hydrogen to produce electrical energy (and/or heat) is the inverse of the water splitting reaction. In this latter case, hydrogen and oxygen react in a fuel cell to produce electrical current, heat, and, as sub-product, water. Hydrogen oxidation can be divided in two sub-reactions: the ORR (happening at the cathode) and the hydrogen oxidation reaction (HOR) (happening at the anode). 

Metals or alloys are typically used to catalyse HER, while OER is efficiently catalysed by metal oxides, whose lattice oxygen atoms can take part in the formation of ^3^O_2_ via the Mars–van Krevelen mechanism. Most of the time, the most efficient metal oxide catalysts for the OER are correlated magnetic materials. A certain material is “correlated” when one can no longer treat the electrons (or better yet, spinons) because of the more influential quantum exchange of non-interacting species. The best strongly correlated materials for OER catalysis have itinerant spin channels, separated in space and associated with dominant ferromagnetic ordering, even above the Curie temperature. The ORR can be catalysed by a variety of different species, though the most used is still platinum metal. Magnetic alloys are the most viable replacement to reduce the need for platinum. Finally, we should note such an interesting phenomenon as spin-crossover (spin isomerism), which is associated with a change in the spin state of the metal atom in some coordination compounds of d- and f-elements under the influence of external factors (temperature, radiation, pressure, etc.). Applications of such compounds rely on the spin-state “switching” effect and include their use as molecular switches and memory storage devices. In this case, complexes with spin crossover, as well as many other metal complexes with organic ligands, can exhibit catalytic activity, which depends on the electronic state of the central atom. Thus, the use of a spin-crossover makes it possible to obtain switchable rather than simple catalysts.

The present Special Issue is entirely dedicated to magnetic interactions in chemistry. The issue welcomes submissions of original research articles and reviews that demonstrate significant advances in the field of magnetism in heterogeneous and homogeneous chemistry alike, such as:

- Structural and spectroscopic characterizations demonstrating novel magnetic orderings or states during catalytic events for novel or already known catalysts and biocatalysts;
- Chemical studies providing solid proof of improvement in catalytic performances by intrinsic and/or extrinsic magnetism. Improvements in catalytic performances may be limited to only reaction rates and/or yields of products or also extended to improved selectivity, improved flow rate, transport properties, and/or adsorption/desorption of substrates on the surface;
- Innovative theoretical treatments, theoretical advances, and new models in understanding and clarifying the role of magnetism in chemical reactions. As mentioned before, new studies have uncovered the connections among the laws that describe the different behaviours of electrons according to their spins, the quantomechanic interactions of exchange and correlation, the formation of intrinsic domains, and the impact of all these factors on catalysis. Such theoretical studies have been and will be helpful to researchers in addressing difficult challenges in the synthesis and characterization of novel magnetic structures with the required optimal magnetic and spin-transport properties. Therefore, scholars providing similar studies are strongly encouraged to be submit their work to this Special Issue;
- State-of-the-art DFT and post-HF computational studies that clarify crucial aspects of magnetism in chemistry. Spin-polarized GGA DFT functionals, corrected by Hubbard term U (among others), are currently the most applied computational methods to investigate magnetism in chemistry, although other methods such as Meta-GGA functionals (which introduce the dependency of the electron density on the kinetic energy), hybrid DFT functionals (which introduce different percentages of exact Hartree–Fock exchange), and dynamical mean field theory (DMFT) are emerging as more accurate alternatives, thanks to the concomitant technological development and improvements in computation seen in the last two decades. Scholars investigating the implementation of correlated post-HF methods, specifically the MP2 method (recently implemented in computational software such as, at the very least, VASP and CP2K), are strongly encouraged to submit their work to this Special Issue. Minor relevance will be given to statistical methods, such as Monte Carlo methods or machine learning approaches that, albeit prospering in solid-state chemistry during the last decade, are deemed by the editors to be less performant in the field of magnetism in chemistry at present (scholars in this field are nonetheless encouraged to submit their work if they deem their work significant);
- New synergistic approaches utilizing theory, computation, and experiments devoted to clarifying crucial aspects of magnetism in chemistry.

In view of the foregoing, this Special Issue of the *International Journal of Molecular Science* is intended as a multi- and interdisciplinary platform on magnetism in chemistry, bringing together various branches of science, primarily biological, coordination, organometallic, theoretical, and computational chemistry and solid-state physics. We hope that the idea of its creation will find wide support from researchers in several fields and will contribute to the emergence of new concepts, such as structural and catalytic innovations, innovative theoretical approaches and/or modern methods of computer analysis, and modelling and synergistic approaches between theory, computation, and experiments. With this Special Issue of the *International Journal of Molecular Science*, we aim to improve the important needed description of magnetism in chemistry.

